# The Phosphatase SHP-2 Activates HIF-1α in Wounds In Vivo by Inhibition of 26S Proteasome Activity

**DOI:** 10.3390/ijms20184404

**Published:** 2019-09-07

**Authors:** Yvonn Heun, Katharina Grundler Groterhorst, Kristin Pogoda, Bjoern F Kraemer, Alexander Pfeifer, Ulrich Pohl, Hanna Mannell

**Affiliations:** 1 Walter Brendel Centre of Experimental Medicine, University Hospital, Ludwig-Maximilians-University, Marchioninistr. 27, 81377 Munich, Germany (Y.H.) (K.G.G.) (K.P.) (U.P.); 2 Biomedical Center, Ludwig-Maximilians-University, Großhaderner Str. 9, 82152 Planegg, Germany; 3 Medizinische Klinik und Poliklinik I, Klinikum der Universität München, LMU, Marchioninistrasse 15, 81377 Munich, Germany; 4 Institute of Pharmacology and Toxicology, Biomedical Center University of Bonn, Sigmund-Freud-Straße 25, 53105 Bonn, Germany; 5 Hospital Pharmacy, Klinikum der Universität München, LMU, Marchioninistrasse 15, 81377 Munich, Germany

**Keywords:** SHP-2, tyrosine phosphatase, HIF-1, 26S proteasome, hypoxia, vascular remodeling, angiogenesis

## Abstract

Vascular remodeling and angiogenesis are required to improve the perfusion of ischemic tissues. The hypoxic environment, induced by ischemia, is a potent stimulus for hypoxia inducible factor 1α (HIF-1α) upregulation and activation, which induce pro-angiogenic gene expression. We previously showed that the tyrosine phosphatase SHP-2 drives hypoxia mediated HIF-1α upregulation via inhibition of the proteasomal pathway, resulting in revascularization of wounds in vivo. However, it is still unknown if SHP-2 mediates HIF-1α upregulation by affecting 26S proteasome activity and how the proteasome is regulated upon hypoxia. Using a reporter construct containing the oxygen-dependent degradation (ODD) domain of HIF-1α and a fluorogenic proteasome substrate in combination with SHP-2 mutant constructs, we show that SHP-2 inhibits the 26S proteasome activity in endothelial cells under hypoxic conditions in vitro via Src kinase/p38 mitogen-activated protein kinase (MAPK) signalling. Moreover, the simultaneous expression of constitutively active SHP-2 (E76A) and inactive SHP-2 (CS) in separate hypoxic wounds in the mice dorsal skin fold chamber by localized magnetic nanoparticle-assisted lentiviral transduction showed specific regulation of proteasome activity in vivo. Thus, we identified a new additional mechanism of SHP-2 mediated HIF-1α upregulation and proteasome activity, being functionally important for revascularization of wounds in vivo. SHP-2 may therefore constitute a potential novel therapeutic target for the induction of angiogenesis in ischemic vascular disease.

## 1. Introduction

The transcription factor hypoxia inducible factor 1α (HIF-1α) is involved in vascular remodeling and angiogenesis [1]. Ischemic cardiovascular disease is characterized by reduced tissue perfusion and reduced tissue oxygen partial pressure (hypoxia), which represent a strong stimulus for HIF-1α activation [2]. HIF-1α induces the expression of several angiogenic genes, such as vascular endothelial growth factor (VEGF), platelet derived growth factor (PDGF) or matrix metalloprotease 2 (MMP-2), which are potent inducers of angiogenesis and arteriogenesis [1,3]. It has therefore been the target of therapeutic strategies to increase tissue perfusion in ischemic limbs [4,5] and to improve wound healing [6,7]. During normoxic conditions, HIF-1α is hydroxylated on prolyl residues within its oxygen-dependent degradation (ODD) domain, leading to von-Hippel-Lindau protein (pVHL) dependent ubiquitinylation and subsequent degradation by the proteasome [8,9]. Hypoxia inhibits the proteasomal degradation of HIF-1α by inhibition of the prolyl hydroxylation domain containing enzymes (PHD), resulting in stabilization of HIF-1α and subsequent accumulation within the cell [8,9]. Additionally, it has been shown that HIF-1α may be degraded by the protease calpain [10].

The tyrosine phosphatase SHP-2 has been demonstrated by us to positively influence angiogenesis in vitro and in vivo [11,12,13] and may constitute an interesting future therapeutic target within this context. In an earlier study we demonstrated that SHP-2 is important for HIF-1α stabilisation and activity during hypoxia, resulting in enhanced hypoxia induced HIF-1α dependent revascularisation of wounds in vivo [12]. Further, we showed that SHP-2 activates the Src kinase upon hypoxia, which in turn influenced HIF-1α prolyl hydroxylation [12]. Finally, we found that the impaired HIF-1α accumulation observed upon SHP-2 inactivation could be rescued by treatment with a PHD inhibitor as well as the proteasome inhibitors MG132 and Epoxomicin [12]. However, while our data indicated that SHP-2 influences HIF-1α accumulation during hypoxia by affecting the activity of the PHD, thus determining the proteasomal degradation of HIF-1α, we did not investigate whether the effect may additionally be caused by regulation of proteasome activity.

The 26S proteasome is responsible for the degradation of ubiquitinylated proteins and consists of a 20S core particle and two 19S regulatory particles [14]. The 20S core particle exhibits three peptidase activities (caspase-like; C-L, trypsin-like; T-L and chymotrypsin-like; CT-L), which are responsible for the cleavage of protein substrates. Ubiquitinylated proteins are recognized and bind to the 19S regulatory particle, which is in addition responsible for the ATP-dependent unfolding of the substrate protein and the opening of the 20S core particle, where degradation occurs [14]. The activity of the 26S proteasome has been shown to be regulated by phosphorylations via threonine/serine kinases as well as tyrosine kinases on several subunits of the 19S and 20S particles [15]. However, not very much is known regarding the role of phosphatases in regulating 26S proteasome activity. Moreover, its regulation during hypoxia still needs to be investigated.

In this study, we investigated the activity of the 26S proteasome during hypoxia and the connection to SHP-2 in endothelial cells in vitro and in hypoxic wounds in vivo. We found SHP-2 to inhibit the 26S proteasome activity in hypoxic cells, as assessed by measuring 26S peptidase activity as well as the accumulation of a HIF-1-ODD-Luc reporter construct. Importantly, we demonstrated that SHP-2 regulates 26S proteasome activity in hypoxic wounds in vivo.

## 2. Results

### 2.1. SHP-2 Inactivation Leads to Increased Proteasome Dependent HIF-1α Degradation during Hypoxia

**Figure 1:**
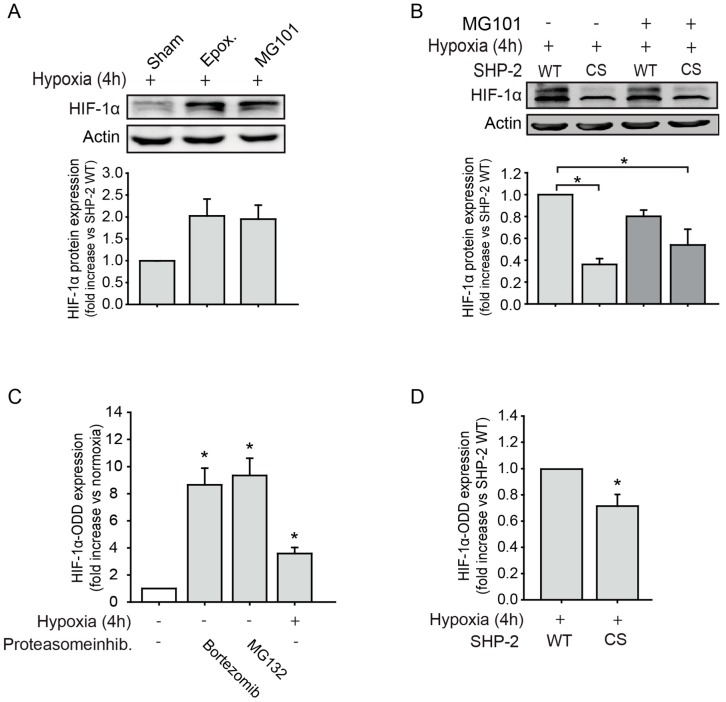
SHP-2 inactivation enhances proteasome dependent hypoxia inducible factor 1α (HIF-1α) degradation in endothelial cells during hypoxia. (**A**) HIF-1α protein levels were increased during hypoxia upon inhibition of the 26S proteasome (Epoxomicin, 10 µM) as well as calpain (MG101, 5 µM) (*n* = 3). Graph underneath blot shows the protein band densities normalized to β-actin. (**B**) Expression of dominant negative SHP-2 (CS) prevents hypoxic HIF-1α protein upregulation, which could not be rescued by calpain inhibition (MG101, 5 µM; * *p* < 0.05; *n* = 3). Graph underneath blot shows the protein band densities normalized to β-actin. (**C**) The reporter construct HIF1-ODD-Luc accumulated upon inhibition of the proteasome (Bortezomib 64 nM; MG132 10 µM) during normoxia as well as under only hypoxia (* *p* < 0.05; *n* = 16–25), confirming the specificity of the reporter constructs. (**D**) Expression of dominant negative SHP-2 (CS) increased HIF-1α degradation by the proteasomal pathway, as detected by lower expression of HIF1-ODD-Luc (* *p* < 0.05, *n* = 17).

As HIF-1α has been shown to be degraded by the proteasome as well as calpain [10], we first investigated whether this is also true in endothelial cells upon hypoxia. Treatment with the specific proteasome inhibitor epoxomicin or the calpain inhibitor MG101, respectively, resulted in an increase in HIF-1α protein accumulation (Figure 1A). We previously observed that SHP-2 inactivation impaired HIF-1α accumulation [12], which was rescued by treatment with proteasome inhibitors. We now additionally investigated the involvement of calpain. As seen in Figure 1B, overexpression of a dominant negative SHP-2 (SHP-2 CS) impaired HIF-1α accumulation under hypoxic conditions compared to cells overexpressing SHP-2 wildtype (WT). Treatment with the calpain inhibitor MG101 could not rescue this effect. Having observed a proteasome dependent [12] but calpain independent degradation of HIF-1α upon SHP-2 inactivation, we next investigated 26S proteasome activity in endothelial cells under hypoxia. For this, we induced the lentiviral expression of a construct containing the oxygen-dependent degradation (ODD) domain of HIF-1α [16], which guides its proteasomal degradation upon ubiquitinylation [3], fused to a luciferase gene (HIF1-ODD-Luc) with simultaneous expression of mCherry, in endothelial cells. The expression of HIF1-ODD-Luc thus inversely correlates with 26S proteasome activity and has been used as a measure of proteasome activity before [16]. First, to test its function in endothelial cells, luciferase activity upon treatment with proteasome inhibitors or hypoxia was measured 72 h after transduction with HIF1-ODD-Luc. As seen in Figure 1C, treatment with the proteasome inhibitors Bortezomib and MG132 as well as hypoxic exposure (4 h) significantly increased the accumulation of HIF1-ODD-Luc, reflecting the inhibition of the 26S proteasome. The transduction of endothelial cells with a control reporter construct lacking the HIF-1α ODD (Ctrl-Luc) showed a strong constitutive expression of luciferase, as expected, which did not differ between normoxic and hypoxic conditions (Figure S1). Expression of dominant negative SHP-2 (CS) impaired HIF1-ODD-Luc accumulation compared to SHP-2 WT expressing cells upon hypoxia, thus demonstrating an increase in 26S proteasome activity (Figure 1D).

### 2.2. SHP-2 Regulates Proteasomal Degradation of HIF-1α in Hypoxic Wounds In Vivo

**Figure 2:**
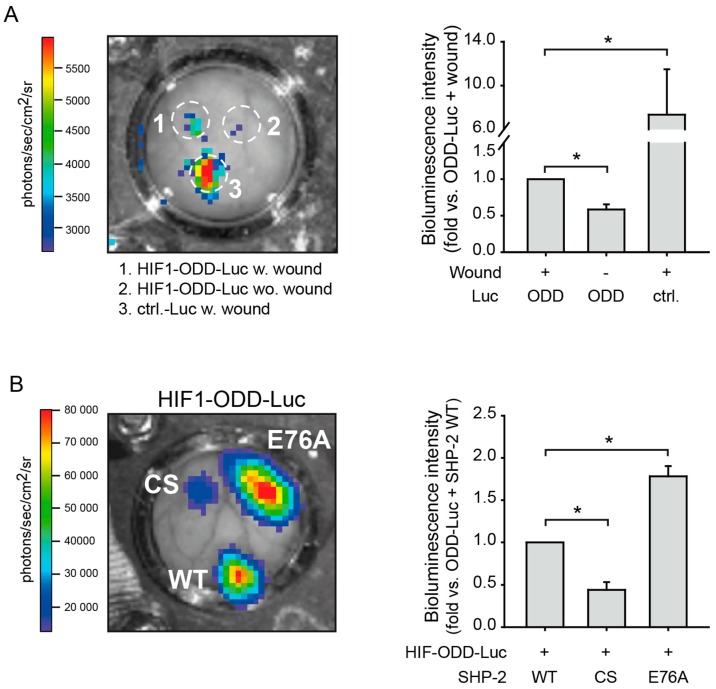
SHP-2 inactivation induces HIF-1α degradation via the proteasome pathway in hypoxic wounds in vivo. (**A**) Wounds in the same dorsal skin fold chamber in mice were simultaneously transduced with HIF1-ODD-Luc or Ctrl-Luc lacking HIF1-ODD using site directed lentiviral magnetic targeting [12]. HIF1-ODD-Luc was expressed in wounds (1) but was degraded by the proteasome in healthy tissue (2), demonstrating the specificity of the lentiviral constructs and that the wounds are hypoxic (* *p* < 0.05; *n* = three animals). (3) Ctrl-Luc lacking the HIF-ODD domain was therefore constitutively expressed in the wound (* *p* < 0.05; *n* = three animals). (**B**) Wounds in the same dorsal skin fold chamber in mice were simultaneously co-transduced with HIF1-ODD-Luc and different SHP-2 constructs. While HIF1-ODD-Luc accumulated in hypoxic wounds upon transduction with SHP-2 WT, expression of dominant negative SHP-2 (CS) impaired this, demonstrating an increased 26S proteasomal activity (* *p* < 0.05; *n* = 3–4 animals). Expression of constitutively active SHP-2 (E76A) further enhanced HIF1-ODD-Luc accumulation, demonstrating enhanced inhibition of 26S proteasome activity (* *p* < 0.05; *n* = 3–4 animals). Wounding was performed the day after implantation of the dorsal skin fold chamber. Transduction of wounds was performed 24h after wounding and measurements of luciferase activity were performed eight days after transduction (see also Figure S2A).

As we previously found SHP-2 inactivation to prevent HIF-1α accumulation and activity in endothelial cells upon hypoxia, resulting in impaired wound healing angiogenesis in vivo [12] and, as we now observed that SHP-2 inactivation increases 26S proteasomal activity under hypoxia in endothelial cells in vitro, we investigated the proteasomal activity in vivo. For this, HIF1-ODD-Luc or Ctrl-Luc were expressed in wounds of the dorsal skin of mice by localized magnetic nanoparticles-assisted lentiviral transduction (Figure S3). By using lentiviruses (LV) coupled to magnetic nanoparticles (MNP) and the application of an external magnetic field, the simultaneous transduction of three individual wounds in the same animal can be achieved [12]. As seen in Figure 2A and Figure S2B, HIF1-ODD-Luc only accumulated in the malperfused wound and not after transduction of healthy tissue, confirming that the wound is hypoxic and that proteasome activity is higher in normoxic tissues. As a positive control, wounds were transduced with Ctrl-Luc, which causes a continuous strong expression of luciferase, as this construct does not contain the HIF-1α ODD domain. Next, we performed co-transductions of individual wounds in the same animal with HIF1-ODD-Luc and the different SHP-2 constructs, to investigate the influence of SHP-2 on proteasome activity in vivo. Whereas the expression of inactive SHP-2 CS in hypoxic wounds significantly inhibited HIF1-ODD-Luc accumulation via increased proteasome activity, introduction of the constitutively active SHP-2 E76A (Glu76 to Ala76) enhanced the HIF1-ODD-Luc protein accumulation compared to SHP-2 WT expressing wounds (Figure 2B and Figure S2C). This indicates that SHP-2 regulates HIF-1α stabilization and accumulation in hypoxic wounds by decreasing 26S proteasome activity.

### 2.3. The Proteasomal Degradation of HIF-1α is Dependent on Src Kinase and p38 MAPK Activation

**Figure 3:**
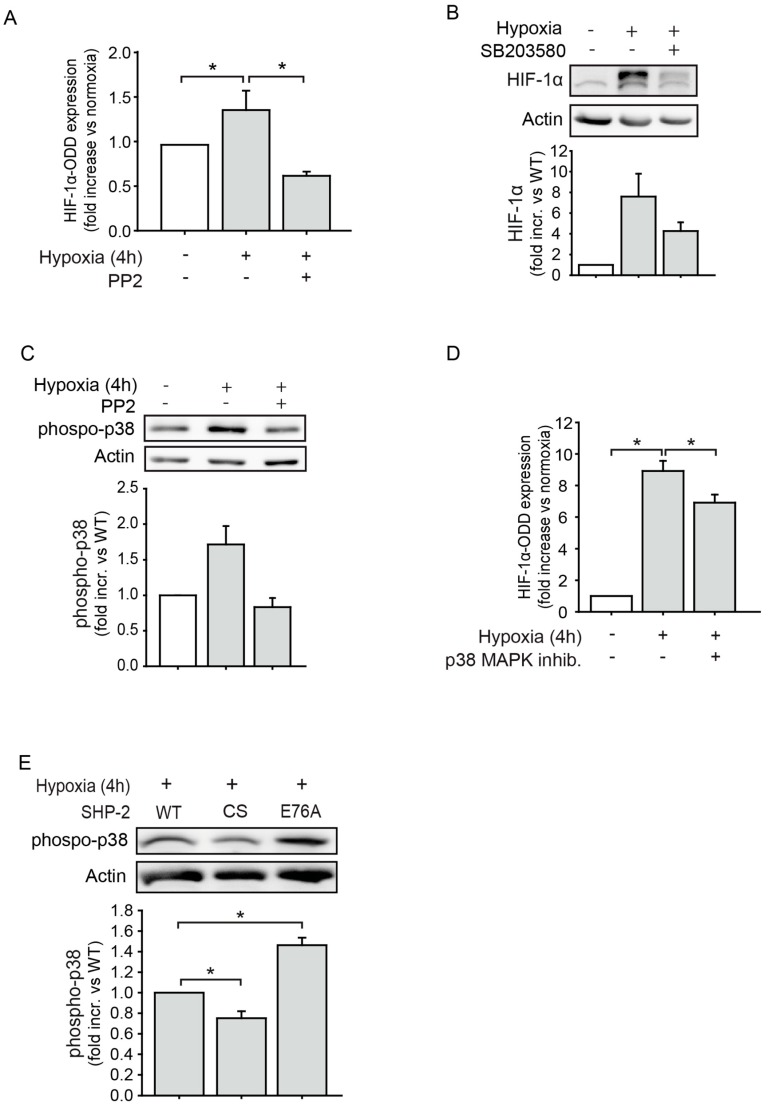
The proteasomal HIF-1α degradation is dependent on Src kinase and p38 mitogen-activated protein kinase (MAPK) signaling. (**A**) Whereas hypoxia inhibited 26S proteasome activity in endothelial cells, as seen by increased expression of HIF1-ODD-Luc, inhibition of Src kinase (PP2, 100 nM) reversed this (* *p* < 0.05; *n* = 9). (**B**) Inhibition of p38 MAPK (SB203580, 10 µM) in endothelial cells impaired hypoxia induced HIF-1α expression (*n* = 6). (**C**) Src kinase inhibition (PP2, 100 nM) reduced hypoxia induced p38 MAPK activation (*n* = 2). (**D**) p38 MAPK inhibition (SB203580, 10 µM) increased 26S proteasome activity, as measured by a lower level of HIF1-ODD-Luc reporter expression (* *p* < 0.05; *n* = 4). (**E**) Expression of dominant negative SHP-2 (CS) impaired hypoxia induced p38 MAPK phosphorylation, whereas expression of constitutively active SHP-2 (E76A) enhanced this compared to SHP-2 WT (* *p* < 0.05; *n* = 4). Graphs underneath blots show the protein band densities normalized to β-actin.

In a former study, we could show that SHP-2 induces HIF-1α expression via a Src kinase dependent mechanism in endothelial cells upon hypoxia [12]. We thus hypothesized that the observed effect of SHP-2 on 26S proteasome activity and HIF-1α stabilization in this study may be mediated by Src as well. To test this, endothelial cells were transduced with the HIF1-ODD-Luc reporter construct and treated with the pharmacological Src inhibitor PP2 upon hypoxia. Whereas hypoxia induced the stabilization, and thus accumulation, of HIF1-ODD-Luc, representing a decrease in proteasome activity, Src inhibition significantly impaired this response (Figure 3A). Src kinases have been demonstrated to induce the activation of p38 MAPK during hypoxia [17]. Thus, we next explored whether this was the case in endothelial cells. The hypoxia induced HIF-1α accumulation was prevented upon inhibition of p38 MAPK (Figure 3B). Moreover, hypoxia induced the phosphorylation and thus activation of p38 MAPK and this was abrogated when treating cells with the Src kinase inhibitor PP2 (Figure 3C). Moreover, treatment with the p38 MAPK inhibitor SB203580 increased proteasome activity, reflected by reduced HIF1-ODD-Luc accumulation (Figure 3D). Finally, the phosphorylation of p38 MAPK was impaired in endothelial cells expressing SHP-2 CS and enhanced in cells expressing the constitutively active SHP-2 E76A compared to the expression of SHP-2 WT upon hypoxia (Figure 3E).

### 2.4. SHP-2 Activity Inhibits the Chymotrypsin-Like Activity of the 26S Proteasome upon Hypoxia

**Figure 4:**
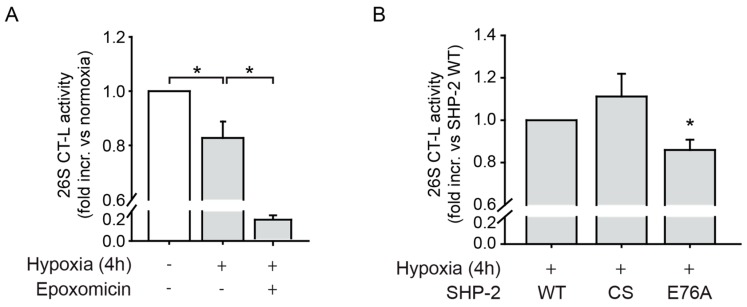
SHP-2 inhibits 26S chymotrypsin-like (CT-L) proteasomal activity during hypoxia. (**A**) The CT-L activity of the 26S proteasome was decreased upon hypoxia (* *p* < 0.05, *n* = 11), as measured by the fluorogenic substrate Suc-LLVY-AMC. Treatment with epoxomicin (10 µM) was used as a positive control and effectively inhibited the CT-L proteolytic activity of the 26S proteasome (* *p* < 0.05; *n* = 3). (**B**) Expression of constitutively active SHP-2 (E76A) also significantly impaired the CT-L activity of the 26S proteasome compared to SHP-2 WT during hypoxia (* *p* < 0.05, *n* = 9), whereas the expression of dominant negative SHP-2 (CS) showed a tendency towards increased CT-L activity (*n* = 9).

As previously published data from our group showed that treatment with epoxomicin and MG132, which are both inhibitors of the CT-L activity of the 26S proteasome [18], rescued the low HIF-1α protein level caused by SHP-2 inactivation [12], we next detected the 26S CT-L proteolytic activity in endothelial cells under hypoxia. We used experimental conditions optimized to investigate proteolytic protease activity of the 26S proteasome as previously described [19]. For this, cells expressing SHP-2 WT were exposed to hypoxia and the chymotrypsin-like (CT-L) activity of the 26S proteasome was assessed by a specific fluorogenic proteasome substrate (Suc-LLVY-AMC). Hypoxia significantly reduced CT-L activity (Figure 4A and Figure S4), and endothelial cells expressing constitutively active SHP-2 E76A exhibited an even lower 26S CT-L activity upon hypoxia compared to SHP-2 WT expressing cells (Figure 4B), indicating a downregulation of the CT-L proteolytic activity of the 26S proteasome.

## 3. Discussion

Vascular remodeling and angiogenesis are important for maintaining tissue perfusion upon ischemia [2]. We previously demonstrated that the tyrosine phosphatase SHP-2 drives hypoxia mediated HIF-1α upregulation, resulting in revascularization of wounds in vivo [12]. Here, we show that this is achieved by SHP-2 dependent inhibition of 26S proteolytic activity via Src kinase/p38 MAPK signalling.

In a former study, we demonstrated that SHP-2 promotes HIF-1α stabilization and activity in endothelial cells during hypoxia as well as revascularization of hypoxic wounds in vivo by increasing HIF-1α activity, resulting in higher expression of VEGF, MMP-2 and PDGF [12]. Moreover, we showed SHP-2 to negatively influence the proteasomal degradational pathway, as the impaired HIF-1α expression seen upon SHP-2 inactivation was rescued by inactivation of the proteasome as well as the PHD [12]. While these results indicate that SHP-2 influences the prolylhydroxylation of HIF-1α and in this way its proteasomal degradation, we further investigated here the mechanisms of SHP-2 and its regulation of proteasomal activity. The data obtained in this study confirm our previous findings and additionally reveal a second mechanism of promoting HIF-1α upregulation by directly affecting 26S proteasomal activity. Inactivation of SHP-2 prevented the cellular accumulation of HIF1-ODD-Luc, which inversely correlated with 26S proteasomal activity, in endothelial cells in vitro. The in vivo relevance of SHP-2 mediated proteasome regulation was confirmed in hypoxic wounds of intact animals using co-transduction of HIF1-ODD-Luc and SHP-2 constructs. Of note, expression of constitutively active SHP-2 further enhanced HIF1-ODD-Luc levels, demonstrating an increased inhibition of the 26S proteasome. This correlates well with the impaired 26S CT-L proteolytic activity measured in cells with constitutive SHP-2 activation. Thus, we conclude that SHP-2 activity negatively affects proteasomal activity during wound healing under hypoxic conditions in vivo.

As we previously found SHP-2 dependent Src kinase activation to be involved in HIF-1α upregulation during hypoxia [12], we now investigated if it is also important for proteasomal activity. Indeed, Src integrity was crucial for 26S proteasomal activity and in addition, was important for activation of the p38 MAPK upon hypoxia. p38 MAPK, in turn, positively affected HIF-1α upregulation while negatively influencing 26S proteasomal activity, a mechanism which was promoted by SHP-2. These results are not only in accordance with but additionally extend the study from Lee et al. performed in HeLa cells, demonstrating that p38 MAPK negatively affects 26S proteolytic activity also in endothelial cells [20]. This inhibition was further shown to be due to the phosphorylation of Thr-273 on the subunit Rpn2 in the 19S regulatory particle [20]. Whether this is the case during hypoxia, remains to be investigated. Posttranslational phosphorylation of the 26S proteasome has repeatedly been shown to regulate its enzymatic activities [15]. Several stimuli, such as stress, metabolic changes, and growth factor signalling have been demonstrated to induce its phosphorylation [15]. Here, we identified hypoxia as a novel stimulus of 26S proteasomal inactivation, particularly the CT-L activity. In addition, we found the caspase-like (C-L) activity to be deprived upon hypoxia (Figure S4). Intriguingly, we measured an increased trypsin-like (T-L) activity during hypoxia, suggesting that the different proteolytic activities of the 26S proteasome may be differentially regulated. One may hypothesize that the differential regulation of activities is involved in substrate specificity of proteasomal degradation. However, more investigations are needed to elucidate the mechanisms behind this. The observed reduction in CT-L and C-L activity, however, is supported by the fact that the 26S proteasome relies on the activity of ATPases in the 19S regulatory particle for substrate de-ubiquitinylation, unfolding and 20S gate opening and activation [15], which do not function upon ATP deprivation, as is the case during hypoxia. Moreover, we observed that inhibition of Src kinase/p38 MAPK signalling rescued proteasomal activation. Additionally, an inhibition of 26S proteasomal activity via direct dephosphorylation of subunits by SHP-2 was not investigated here and has to be the focus of further studies. However, this is per se possible, as Zong et al. demonstrated the phosphatase PP2A to negatively affect 20S proteolytic activity via dephosphorylation [21]. Nevertheless, this is to our knowledge the first study to report a regulation of 26S proteolytic activity by SHP-2.

**Figure 5:**
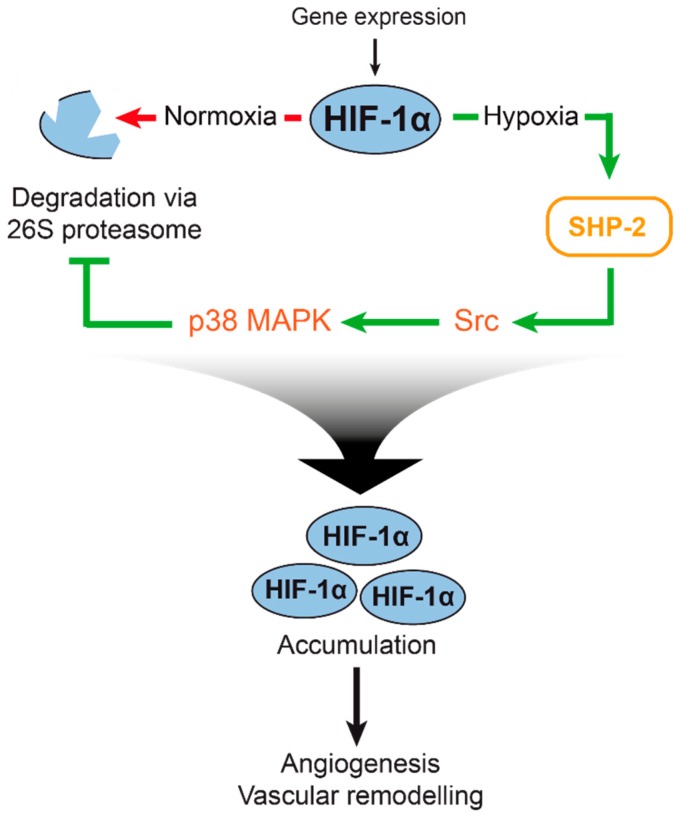
Illustration of 26S proteasome regulation and HIF-1α accumulation by SHP-2 in hypoxia. HIF-1α is constitutively expressed but degraded by the proteasomal pathway during hypoxia (left). Upon hypoxia (right), the degradation of HIF-1α is inhibited. In our previous study, we found SHP-2 to influence the prolyl hydroxylation of HIF-1α, which targets it for degradation [12]. In this study, we found a second mechanism of SHP-2 mediated HIF-1α upregulation: SHP-2 positively affects Src kinase and p38 MAPK activity during hypoxia, which in turn negatively influences the activity of the 26S proteasome. As a consequence, HIF-1α is stabilized and accumulates in the cell, promoting hypoxic angiogenesis and vascular remodeling.

In summary, we were able to further characterize the mechanism behind the regulation of hypoxia mediated HIF-1α upregulation by SHP-2, which is essential for revascularisation of malperfused wounds. Moreover, we demonstrate for the first time that the 26S proteasomal activity is regulated by SHP-2 during hypoxia in vitro and is functionally relevant in vivo. We show that SHP-2 not only inhibits the proteasomal degradation pathway by influencing HIF-1α prolylhydroxylation [12] but in addition by directly inhibiting 26S proteolytic activity via p38 MAPK (for a summary of our findings, see Figure 5). We thus believe that SHP-2 is important for HIF-1α upregulation in vitro and in wounds in vivo by inhibition of the proteasomal pathway via activation of Src kinase/p38 MAPK signalling. Together with our previous results, this regulation may be achieved by redundant and or additive pathways, involving external regulators of the proteasome (PHD and pVHL activation), and directly via the CT-L activity of the 26S proteasome. Finally, our findings confirm SHP-2 to be essential for hypoxic HIF-1α upregulation in vivo. SHP-2 may therefore constitute a novel therapeutic target in ischemic vascular disease, aiming for the revascularization of ischemic tissues.

## 4. Materials and Methods

### 4.1. Antibodies and Chemicals

Rabbit phospho-p38 MAPK (Thr180/Tyr182) (D3F9) XP™ (#4511) and β-Actin (13E5) antibodies were from Cell Signaling Technology, Frankfurt am Main, Germany. HIF-1α clone H1α67, HIF-1α clone EP1215Y, anti-mouse and -rabbit horseradish peroxidase-conjugated secondary antibodies were from Merck Millipore, Darmstadt, Germany. Src-Inhibitor PP2 was purchased from Sigma-Aldrich (#P0042), Darmstadt, Germany. p38 MAPK inhibitor (SB203580), MG132 and MG101 were from Tocris, Wiesbaden-Nordenstadt, Germany. Bortezomib and Epoxomicin were from Calbiochem. All other chemicals were from Sigma-Aldrich, Darmstadt, Germany.

### 4.2. Human Microvascular Endothelial Cell (HMEC) Culture

Human dermal microvascular endothelial cells (HMEC) [22] were cultivated as described earlier [23]. In detail, HMEC were cultivated in DMEM (Sigma-Aldrich, Darmstadt, Germany) containing 10% fetal calf serum (Biochrom, Berlin, Germany) and 1% Penicillin-Streptomycin (Sigma-Aldrich, Darmstadt, Germany) and kept in an incubator with 5% CO_2_ at 37 °C.

### 4.3. Lentiviral Constructs and Transductions

Wild type (WT) SHP-2 and catalytically inactive mutant SHP-2 CS (Cys459 to Ser459) plasmid vectors were kind gifts from the Bennett laboratory [24]. The constitutively active SHP-2 E76A (Glu76 to Ala) was generated as previously described [12]. The lentiviral constructs containing the above mentioned cDNAs were generated as described earlier [12] and lentiviral particles were produced as described elsewhere [25]. Flow cytometry of transduced HEK293T cells was used to determine the biological titer as previously described [25]. The FUW-ODD-Luc-mCherry lentiviral plasmid (referred to as HIF1-ODD-Luc in this manuscript) and the control vector FUW-Luc-mCherry (referred to as Ctrl-Luc in this manuscript) were kindly provided by Kimbrel et al. [16] and packaged into lentiviral particles as previously described [25]. The ODD-Luc insert encodes a reporter fusion protein consisting of the oxygen-dependent domain (ODD) from hypoxia-inducible factor 1a (HIF-1a) and firefly luciferase, inversely reflecting proteasomal activity, with simultaneous expression of mCherry after a self-cleavage 2A site. Lentiviral transduction of HMECs was carried out using a multiplicity of infection (MOI) of five. Lentiviral particles were diluted in Hank’s solution and applied onto subconfluent HMEC. After incubation for 4–6 h at 37 °C, culture medium was added. The next day the medium was changed, and cells were left 72 h before assaying.

### 4.4. Hypoxia Treatment

For hypoxia treatment, HMECs were incubated in cultivation media in a hypoxia chamber (Cell Systems, Troisdorf, Germany) at pO_2_ 8 ± 2 mmHg equivalent to a O_2_ concentration of 1 ± 0.2% for 4 h as previously described [26]. To reach these experimental hypoxic conditions, the chamber was flooded for four minutes with 15–20 L/min with an anoxic gas mixture (5% CO_2_, 95% N_2_). After hypoxia, the media was discarded, and cells quickly washed once with phosphate buffered saline and lysed for further processing.

### 4.5. In Vitro 26S Proteasome Activity

The 26S CT-L proteasome activity was measured as previously described [19]. In detail, HMEC were subjected to hypoxia (4 h), rinsed with cold phosphate buffered saline supplemented with calcium (PBS+) and lysed at 4 °C with lysis buffer (1mM DTT, 1× Roche PhosphoStop Tablet in homogenizing buffer containing 20 mM HEPES, 1 mM MgCl_2_, 150 mM NaCl, and 0.5 mM EDTA). 10 µg protein in assay buffer containing 1mM DTT, 50 µM ATP and 100 µM Suc-LLVY-AMC (R&D systems, Wiesbaden, Germany) in homogenizing buffer was measured at 37 °C with excitation wavelength 339 nm and emission wavelength 439 nm for 2 h.

### 4.6. In Vitro Luciferase Assay

HMEC were seeded in 24-well plates and transduced the following day. Indicated inhibitor treatments and hypoxia was performed 72 h post transduction and cells were lysed on ice for 30 min (250 mM Tris base pH7.8, 0.1% Triton-X). Then, 10 µL of cell lysate were transferred to a black 96-well plate in triplicates and 100 µL luciferin assay buffer (60 mM DTT, 10 mM MgSO_4_, 1 mM ATP, 25 mM Glycil-Glycin, 0.3 mM D-Luciferin) were added to each well. Bioluminescence was detected using the Spectrafluor (Tecan, Männedorf, Switzerland) with a one-second integration time and normalized to protein concentrations.

### 4.7. In Vivo Transduction and Luciferase Imaging

Animal studies were conducted in accordance with the German animal protection law and approved by the district government of upper Bavaria (Regierung von Oberbayern, approval reference number AZ55.2-1-54-2532-172-13). The investigation conforms to the Guide for the Care and Use of Laboratory Animals published by the US National Institutes of Health (NIH Publication No. 85-23, revised 1996). 16–20-week old male and female C57 BL6/J mice (Charles River) were anesthetized (5 mg/kg midazolam, 0.5 mg/kg medetomidin, and 0.05 mg/kg fentanyl) and the dorsal skinfold chamber was implanted as described before [27]. Wounds were introduced using a hot probe as previously described 24 h after dorsal skinfold chamber implantation [12]. Proteasomal activity in avascular wounds in the dorsal skin of mice was detected by localized magnetic nanoparticles-assisted transduction of individual wounds in the same animal with HIF1-ODD-Luc (FUW-ODD-Luc-mCherry) and Ctrl-Luc (FUW-Luc-mCherry) lentiviral vectors as well as co-transductions of with the ODD-Luc vector and SHP-2 WT, CS and E76A lentiviral vectors, respectively, as previously described [12]. Luciferase activity was detected eight days after transduction by application of 3 mg/mL D-Luciferin directly to the imaging window of the dorsal skinfold chamber. Bioluminescence was imaged using the IVIS^®^ spectrum in vivo imaging system from PerkinElmer at medium binning and exposure times between five and 10 minutes. Signal intensities were quantified using the Fiji software.

### 4.8. Immunoblotting

Cell lysates were prepared and subjected to SDS-PAGE followed by western blotting as previously described [28]. Protein band intensities were measured using the Hokawo software (Hamamatsu, Herrsching, Germany) and normalized to the respective β-Actin protein bands, which was used as equal loading control.

### 4.9. Statistical Analysis

Data are presented as means ± SEM. Statistical analyses were performed with Sigma Plot 10.0. The Student’s t-test was used for comparisons between two groups of normal distributed data, rank-sum test was performed for comparisons of two groups of not normally distributed data. The one-way analysis of variance (one-way ANOVA) was performed for multiple comparisons. Differences were considered significant at an error probability level of *p* < 0.05.

## Supplementary Materials

Supplementary materials can be found at https://www.mdpi.com/1422-0067/20/18/4404/s1.

## Abbreviations

Ctrl-Luc	Lentiviral control luciferase reporter construct lacking the HIF-1α ODD
HIF1-ODD-Luc	Lentiviral construct containing the oxygen-dependent degradation (ODD) domain of HIF-1α fused to luciferase
HMEC	Human microvascular endothelial cells
SHP-2	Src homology domain containing tyrosine phosphatase 2
SHP-2 WT	SHP-2 wildtype construct
SHP-2 CS	Dominant negative SHP-2 construct where Cys459 was exchanged to Ser459
SHP-2 E76A	Constitutively active SHP-2 construct where Glu76 was exchanged to Ala76
