# Supplementary material for: The Phosphatase SHP-2 Activates HIF-1α in Wounds In Vivo by Inhibition of 26S Proteasome Activity
Source: Int J Mol Sci. 2019 Sep 7;20(18):4404. doi: 10.3390/ijms20184404 (PMC6769879; doi:10.3390/ijms20184404)
Supplement: Supplementary file 1 [file ijms-20-04404-s001.pdf]

Supplementary figures

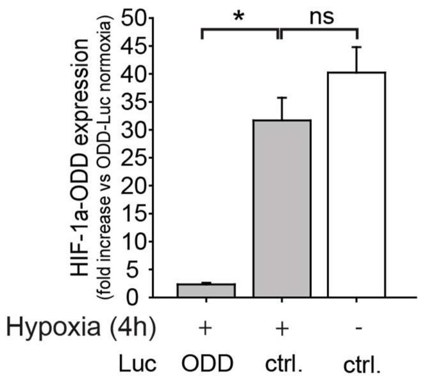

Figure S1. Expression on Ctrl-Luc and HIF1-ODD-Luc in normoxia and hypoxia

Endothelial cells were transduced with lentiviral HIF1-ODD-Luc and Ctrl-Luc (lacking the HIF1-ODD) reporter constructs. Expression of the Ctrl-Luc reporter construct did not differ between normoxia and hypoxia (ns: not significant), and was higher than HIF1-ODD-Luc, as Ctrl-Luc is lacking the ODD and thus is not degraded upon normoxia (4h) (*$p<0.05$, $n=7$).

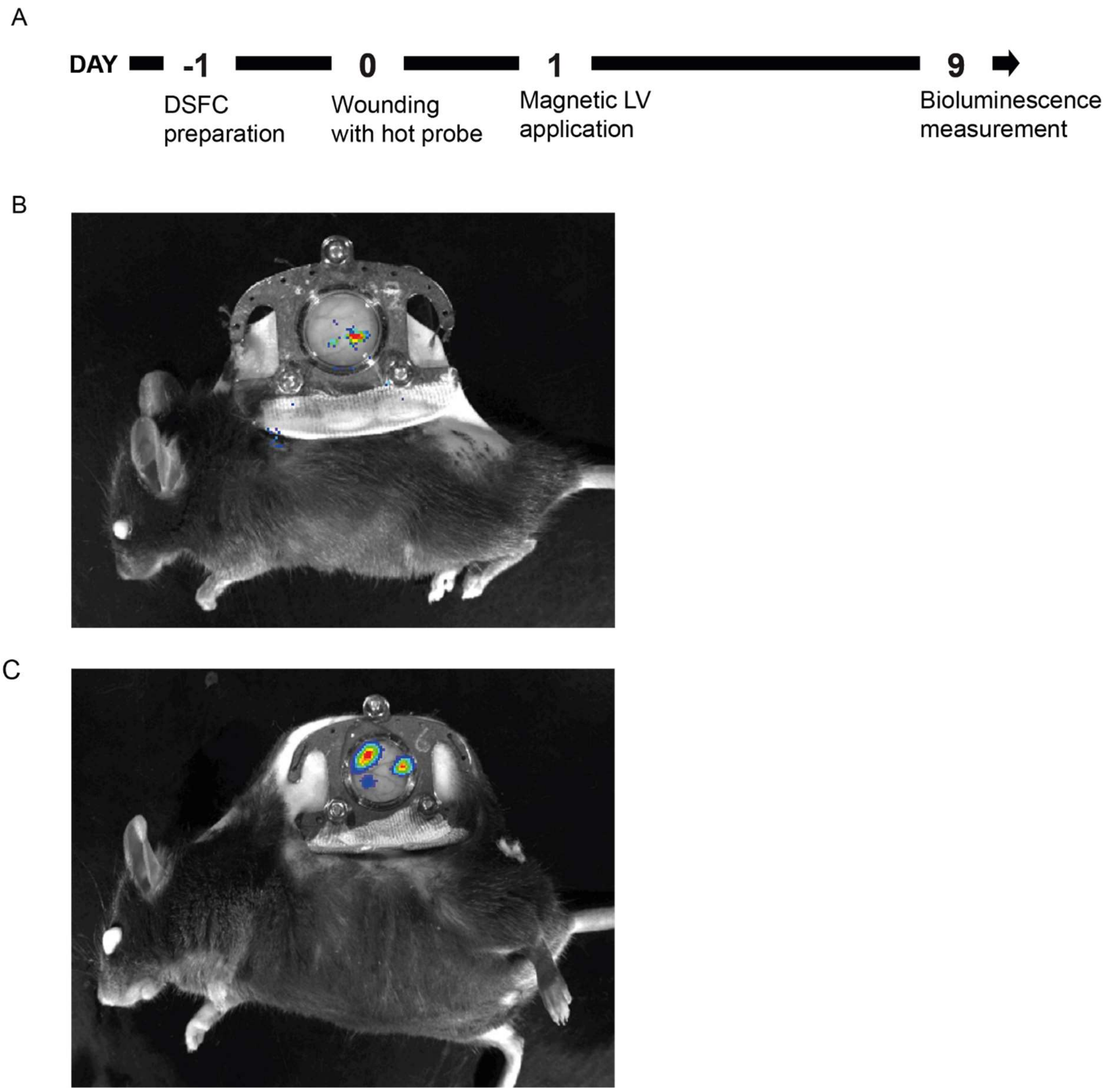

Figure S2. Site specific magnetic targeting of lentiviral constructs in wounds *in vivo*

A) Time line of *in vivo* experiments: The dorsal skinfold chamber (DSFC) was implanted (Day -1) followed by introduction of wounds in the dorsal skin the next day (Day 0). 24h after wounding, lentiviral (LV) transduction using site directed magnetic targeting [12] was performed (Day 1). 8 days (Day 9) after transduction, bioluminescence was detected. B) Bioluminescence picture of whole mouse from Fig. 2A in the main manuscript with wounds in the same dorsal skin fold chamber, which were simultaneously transduced with HIF1-ODD-Luc or Ctrl-Luc using site directed lentiviral magnetic targeting. C) Bioluminescence picture of whole mouse from Fig. 2B in the main manuscript with wounds in the same dorsal skin fold

chamber co-transduced with HIF1-ODD-Luc and different SHP-2 constructs. The pictures show transgene expression at the target sites (wounds) only.

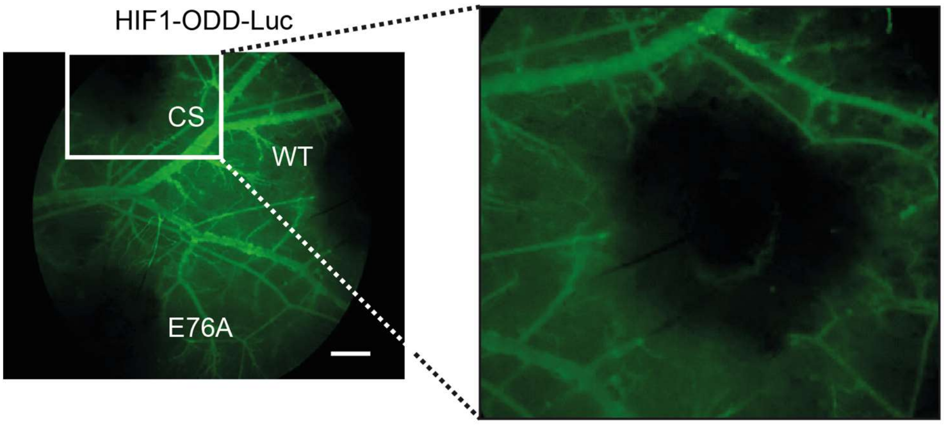

Figure S3. Wounds in the dorsal skin fold chamber of mice

Representative photos of wounds in the dorsal skin fold chamber at day of transduction (1 day after wounding), which all were transduced with HIF1-ODD-Luc in combination with either SHP-2 WT, CS or E76A by localized magnetic nanoparticle-assisted lentiviral gene delivery [12]. FITC-Dextran (150kDa, 20µl; green) was injected intravenously via the tail vein to visualize the vascular network around the wounds, followed by imaging with an intravital microscope. Bar in photos represent 500µm.

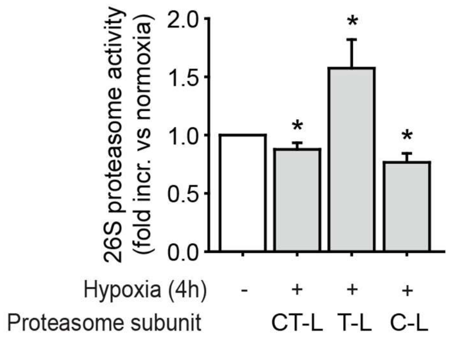

Figure S4. 26S proteasome activities during hypoxia

The chymotrypsin-like (CT-L) and the caspase-like (C-L) activities of the 26S proteasome were impaired upon hypoxia (*$p<0.05$; n=11-14), whereas the trypsin-like activity (T-L) was increased (*$p<0.05$; n=13) compared to the respective activity during normoxia. Values for the CT-L activity are the same as displayed in Figure 4. Activity values (CT-L, T-L and C-L) are all normalized to their respective activity during normoxia. The proteolytic activities were assessed using the specific fluorogenic substrates Suc-LLVY-AMC (CT-L), Boc-LSTR128 (T-L) (Bachem) and Z-LLE-AMC (C-L) (Boston Biochem), as previously described [19].
